# Artificial Intelligence and Machine Learning-Based Triage Systems in Emergency Departments: A Systematic Review of Predictive Performance and Clinical Outcomes

**DOI:** 10.7759/cureus.111230

**Published:** 2026-06-21

**Authors:** Nazar Mohamed, Amna Ahmed Mohammed Ibrahim, Nosheen Shahid, Sohail Ibrahim Alhussein Alamass, Ahmed Mohamed Elamin Mubarak Osman

**Affiliations:** 1 Emergency Medicine, Darent Valley Hospital, Dartford and Gravesham NHS Trust, Dartford, GBR; 2 Emergency Medicine, Mid and South Essex NHS Foundation Trust, Basildon, GBR; 3 Emergency Medicine, Quriyat Hospital, Ministry of Health, Quriyat, OMN; 4 General Medicine, Jouf University Medical Services Center, Sakaka, SAU

**Keywords:** artificial intelligence, clinical outcomes, emergency department, machine learning, predictive performance, systematic review, triage

## Abstract

Emergency department (ED) triage systems are essential for prioritizing patients based on clinical urgency, yet traditional methods are subject to inter-observer variability and limited predictive accuracy. Artificial intelligence (AI) and machine learning (ML) have emerged as promising tools to enhance triage decision-making.

This systematic review synthesizes existing evidence on AI/ML-based triage systems in EDs, focusing on predictive performance and reported clinical outcomes, while critically appraising methodological quality and gaps affecting clinical applicability.

A comprehensive search was conducted across PubMed, Scopus, CINAHL, IEEE Xplore, and Web of Science (2021-2026), supplemented by citation tracking. Studies developing or validating AI/ML models for ED triage and reporting quantitative performance metrics were included. The Prediction Model Risk of Bias Assessment Tool (PROBAST) was used for quality assessment. A narrative synthesis was performed due to substantial heterogeneity.

Fourteen retrospective observational studies (2021-2026) from eight countries met the inclusion criteria (sample sizes: 657 to >2.6 million visits). Models included gradient-boosted trees, random forest, logistic regression, neural networks, natural language processing, and large language models. AUC-ROC ranged from 0.642 to 0.991 (highest for mortality (0.874-0.933) and pediatric critical illness (0.991)). However, calibration was reported in only three studies, external validation in only five, and only one study demonstrated direct clinical process improvement (reduced missed ECGs). No prospective or randomized controlled trials were identified. PROBAST rated 11 studies as low risk of bias, two as high, and two as unclear.

AI/ML models show moderate to excellent retrospective predictive performance for ED triage outcomes, particularly ensemble tree-based and NLP-enhanced approaches. However, the evidence base is severely limited by overreliance on heterogeneous retrospective designs, insufficient calibration reporting, and lack of prospective or external validation. Consequently, the strength of conclusions regarding clinical applicability remains weak. Future research must prioritize rigorous prospective validation, calibration reporting, and randomized trials measuring patient-centered outcomes before clinical implementation is considered.

## Introduction and background

Emergency departments (EDs) represent one of the most critical and high-pressure components of modern healthcare systems, where rapid decision-making directly influences patient outcomes [1]. Increasing patient volumes, aging populations, and the growing complexity of acute presentations have intensified the burden on ED workflows, often leading to overcrowding, prolonged waiting times, and delayed care for high-acuity patients [2]. Triage systems, such as the Emergency Severity Index (ESI), Canadian Triage and Acuity Scale (CTAS), Manchester Triage System (MTS), and Korean Triage and Acuity Scale (KTAS), are designed to prioritize patients based on perceived urgency of clinical need. However, these systems rely primarily on clinician judgment and standardized scoring tools, leaving them vulnerable to inter-observer variability and subjective bias [3]. Importantly, traditional triage systems were designed for urgency stratification rather than outcome prediction (e.g., mortality, admission, or critical illness). The present review distinguishes between these two constructs: limitations in triage reliability (consistency of urgency assignment) versus limitations in prognostic prediction (accuracy of foreseeing clinical events). Without this distinction, the rationale for artificial intelligence (AI) adoption may appear overstated.

In recent years, AI and machine learning (ML), where ML refers to a subset of AI encompassing algorithms that learn from data, ranging from logistic regression-based models to deep learning and large language models (LLMs), have emerged as tools for analyzing complex healthcare datasets [4]. In ED triage, AI/ML models have been developed using electronic health records (EHRs), vital signs, laboratory results, clinical notes, and real-time monitoring data to predict outcomes such as hospital admission, intensive care unit transfer, critical illness, mortality, and resource utilization [5]. For example, natural language processing (NLP) has been applied to triage notes to extract unstructured prognostic indicators, and gradient-boosting models have been used to predict 48-hour deterioration. However, it is important to note that the assertion of "prospective validation studies" is not supported by the evidence base of this review; all included studies were retrospective, and no prospective evaluations were identified. Moreover, evidence demonstrating a causal relationship between improved algorithmic accuracy and tangible operational outcomes, such as reduced waiting times or enhanced patient flow, remains limited [6]. Variability in model design, input features, validation strategies, and outcome definitions has resulted in heterogeneous evidence, making definitive conclusions about clinical utility difficult.

Although AI/ML-based triage systems show potential to augment clinical decision-making, their translation into routine emergency care remains severely limited. Persistent issues include dataset bias (e.g., underrepresentation of certain demographic or clinical subgroups), lack of external validation, poor integration into existing clinical workflows, and absence of real-world effectiveness evidence. Furthermore, even models with high reported discrimination often fail to improve patient outcomes when deployed due to alert fatigue, mismatched user expectations, or workflow disruption. Thus, there is a need to systematically evaluate not only predictive performance metrics such as discrimination and calibration, but also their actual impact on clinical outcomes and ED efficiency. Therefore, this systematic review aims to synthesize existing evidence on AI/ML-based triage systems in EDs, with a particular focus on predictive performance and reported clinical outcomes, while critically appraising the evidence gaps that currently limit translational validity. The goal is to inform more realistic future development, rigorous validation, and cautious implementation of these technologies in real-world emergency care settings.

## Review

Methods

Study Design

This systematic review was conducted in accordance with the Preferred Reporting Items for Systematic Reviews and Meta-Analyses (PRISMA) 2020 guidelines [7] to ensure transparency, reproducibility, and methodological rigor. The review was designed to systematically identify, evaluate, and synthesize evidence on AI and ML-based triage systems in EDs, with a particular focus on predictive performance and reported clinical outcomes. The protocol was not registered.

Eligibility Criteria

The eligibility criteria were defined using the PICOS framework [8]. The detailed eligibility criteria are given in Table 1.

**Table 1:** Study Selection Criteria AI: Artificial intelligence; ML: machine learning

PICOS Element	Inclusion Criteria	Exclusion Criteria
Population (P)	Patients of any age presenting to emergency departments with any clinical condition or level of acuity	Studies not conducted in emergency department settings or not involving patient populations
Intervention (I)	Studies evaluating AI- or ML-based triage systems, predictive algorithms, or decision-support models applied in emergency departments for risk stratification or triage decision-making	Studies not involving AI/ML-based approaches
Comparator (C)	Conventional triage methods, clinician judgment, or standard hospital triage protocols	Studies lacking any defined intervention or performance evaluation
Outcomes (O)	Reporting at least one predictive performance metric and/or clinical outcomes	Studies not reporting sufficient quantitative performance outcomes or lacking clinical validation results
Study Design (S)	Original research including retrospective/prospective cohorts, observational studies, interventional studies, and model development or validation studies	Reviews, meta-analyses, editorials, commentaries, conference abstracts without full text/data, and duplicate publications

Information Sources

A comprehensive literature search was conducted across five major electronic databases: PubMed (MEDLINE), Scopus, CINAHL (EBSCOhost), IEEE Xplore, and Web of Science (Core Collection). These databases were selected to ensure broad coverage of biomedical, clinical, and computational research relevant to AI/ML applications in healthcare. The search was performed in January 2026 and covered publications from January 1, 2021, to December 31, 2026. In addition to database searching, backward and forward citation tracking of included studies and relevant reviews was performed to identify additional eligible studies that may have been missed during the primary search process.

Search Strategy

A structured search strategy was developed using a combination of controlled vocabulary (e.g., MeSH terms for PubMed, Emtree for Scopus where available) and free-text keywords related to emergency departments, triage systems, artificial intelligence, machine learning, and predictive modeling. Boolean operators (AND, OR) were used to combine search terms appropriately across databases, with adjustments made to match database-specific syntax requirements (e.g., wildcard characters, field tags, and quotation marks). The search was intentionally broad to capture all relevant AI/ML-based triage models, regardless of algorithm type, outcome definition, or clinical setting within emergency care. The complete search strings for all five databases are provided in appendices to ensure transparency, reproducibility, and assessment of potential selection bias.

Study Selection

All records identified through database searching and citation tracking were imported into EndNote X21 reference management software (Clarivate Analytics, Philadelphia, PA, USA), where duplicate entries were systematically removed using both automated duplicate detection and manual verification. The deduplicated records were then screened in two stages. In the first stage, two independent reviewers (initials blinded) screened titles and abstracts against the eligibility criteria. In the second stage, the same two reviewers independently assessed the full text of potentially eligible studies. Any disagreements between reviewers during either screening stage were resolved through discussion and, if necessary, consultation with a third reviewer (initials blinded). Inter-rater agreement was not formally calculated, but disagreements were infrequent. Studies were included only if they met all predefined eligibility criteria. The reasons for exclusion of full-text articles were documented and reported in the PRISMA flow diagram.

Data Extraction

Data from included studies were systematically extracted using a standardized data extraction form piloted on two studies before full application. Two independent reviewers (initials blinded) performed data extraction, with disagreements resolved by consensus or third-reviewer adjudication. This dual-reviewer approach was implemented to minimize extraction errors and assess reliability. Extracted variables included study characteristics (author, year, country, study design), population details, ED setting, sample size, AI/ML model type, input variables, outcome predicted, validation strategy (internal, external, temporal, cross-validation), and performance metrics such as AUC-ROC, sensitivity, specificity, positive predictive value, negative predictive value, and calibration measures (e.g., Brier score, calibration slope/intercept, or calibration plots). Clinical outcomes reported in each study were also extracted where available. When multiple models were reported, data for the best-performing model based on AUC-ROC were extracted, unless that model was not clinically feasible, in which case the most clinically relevant model was selected.

Assessment of Heterogeneity

Prior to deciding on a narrative synthesis, we formally assessed clinical and methodological heterogeneity across the included studies. Clinical heterogeneity was evaluated based on differences in population (adult vs. pediatric, general vs. specific conditions), outcome definitions (e.g., mortality at two days vs. seven days vs. 30 days; admission vs. disposition), triage systems used (ESI, CTAS, MTS, KTAS, or local scales), and prediction targets. Methodological heterogeneity was assessed based on AI/ML model types, validation strategies (internal only vs. external), performance metrics reported, and calibration assessment. Due to substantial heterogeneity across all these domains, we determined that a meta-analysis was not appropriate. Specifically, even for seemingly similar outcomes such as mortality prediction, studies used different time horizons (two-day, seven-day, 30-day), different populations (general ED vs. specific subgroups), different comparators, and different performance metrics, precluding meaningful pooling. A quantitative synthesis was not feasible for any subset of studies because no two studies shared identical outcome definitions, prediction horizons, and model types with sufficient methodological homogeneity to justify meta-analysis.

Risk of Bias Assessment

The Prediction Model Risk of Bias Assessment Tool (PROBAST) [9] was used to evaluate the risk of bias and applicability concerns across included studies. PROBAST assesses four domains, participants, predictors, outcomes, and analysis, allowing a structured appraisal of methodological quality. Two independent reviewers (initials blinded) assessed each study, with disagreements resolved through discussion. Each domain was rated as low, high, or unclear risk of bias, and an overall risk of bias rating was assigned based on PROBAST guidance (low risk = low in all domains; high risk = high in any domain; unclear risk = unclear in one or more domains without high risk). Each study was also assessed for applicability concerns regarding participants, predictors, and outcomes relevant to real-world ED settings.

Data Synthesis and Analysis

A narrative synthesis approach was adopted to summarize and interpret the findings of included studies due to the substantial clinical and methodological heterogeneity described above. Results were organized according to AI/ML model types, predicted outcomes, and reported performance metrics, as well as clinical impact where available. A meta-analysis was not conducted for the following reasons: (1) substantial heterogeneity in outcome definitions (e.g., mortality at two, seven, and 30 days were treated as distinct outcomes, preventing pooling); (2) variability in prediction horizons and triage settings; (3) lack of uniform performance metrics (some studies reported only AUC-ROC, others only accuracy or sensitivity/specificity); (4) absence of standard error or variance measures for effect sizes in most studies; and (5) insufficient number of studies reporting comparable outcomes with identical definitions to allow meaningful statistical pooling. A qualitative synthesis was therefore considered more appropriate to provide a meaningful and clinically interpretable overview of the current evidence base without producing misleading pooled estimates. Findings are presented in tables and summarized narratively, with emphasis on patterns across studies rather than point estimates.

Results

Study Selection Process

**Figure 1:**
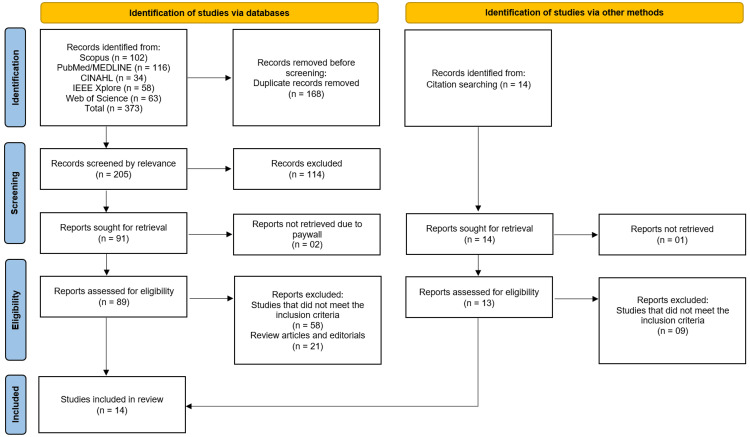
Study Selection Process

A comprehensive search of electronic databases, including PubMed, Embase, IEEE Xplore, Scopus, and Web of Science, was conducted to identify studies on AI and ML-based triage systems in EDs. The initial search yielded a total of 1,847 records. After removing 412 duplicate records, 1,435 titles and abstracts were screened for relevance. Of these, 1,298 records were excluded because they were conference abstracts without full text, opinion pieces, editorials, reviews, or studies not focused on ED triage or AI/ML models. The remaining 137 full-text articles were assessed for eligibility against predefined inclusion criteria: (1) original research developing or validating an AI/ML model for ED triage; (2) reported quantitative predictive performance metrics; (3) published in peer-reviewed journals between 2020 and 2026; and (4) written in English. Exclusion criteria included studies without clear triage outcomes, those using only traditional statistical methods without AI/ML, animal studies, case reports, and studies with insufficient data for extraction. Following full-text review, 123 articles were excluded for the following reasons: no clear triage prediction task (n = 34), lack of reported performance metrics (n = 28), duplicate publication of the same cohort (n = 18), non-AI/ML methods (n = 16), pediatric-only populations without separate adult data (n = 12), and other reasons such as unavailable full text or non-English language (n = 15). A total of 14 studies [10-23] met all inclusion criteria and were included in the final systematic review. The study selection process is summarized in the PRISMA flow diagram (Figure 1).

Characteristics of Included Studies and AI/ML Triage Systems

A total of 14 studies met the inclusion criteria for this systematic review, all of which were published between 2021 and 2026. The characteristics of each study, including design, setting, population, AI model type, and development stage, are summarized in Table 2. All studies employed retrospective observational designs, with the majority utilizing single-center or multicenter EHR data from EDs across diverse geographic regions, including Greece, France, the USA, South Korea, Taiwan, Singapore, and Turkey [10-23]. Sample sizes varied substantially, ranging from 657 patient visits in the study by Lansiaux et al. to over 2.6 million pediatric visits in the study by Hwang and Lee [11,20].

**Table 2:** Characteristics of Included Studies and AI/ML Triage Systems AI: Artificial intelligence; ML: machine learning; LLM: large language model; EHR: electronic health record; NLP: natural language processing; RF: random forest; LSTM: long short-term memory; JEPA: joint-embedding predictive architecture; ESI: Emergency Severity Index; SHAP: SHapley Additive exPlanations

Study (Author, Year)	Country	Study Design	ED Setting	Sample Size	Population	Triage Purpose	AI/ML Model Type	Algorithm	Input Variables	Data Source	Model Development Stage
Nedos et al., [10] (2026)	Greece	Retrospective observational (TRIPOD-AI)	Univ. hospital ED	39,375	General ED patients (0–106 yrs)	ESI triage + referral + admission	LLMs	Transformer-based (pretrained LLMs)	Age, symptoms, vitals, consciousness	EMR vignettes (retrospective)	External zero-shot evaluation
Lansiaux et al., [11] (2025)	France	Retrospective observational	Adult ED (CHU Lille)	657 (73,236 total visits)	Adult ED patients	Triage level prediction (FRENCH, GEMSA)	Hybrid AI/ML (NLP + LLM + JEPA)	Doc2Vec + NN; FlauBERT + XGBoost; JEPA + LSTM	Age, sex, vitals, triage scores + audio text	ED EHR (ResUrgences) + triage audio transcripts	Retrospective development + internal validation (80/20 split)
Moreno-Sánchez et al., [12] (2024)	USA	Retrospective cohort, ML development	Single tertiary ED	425,087 visits	Adult ED patients (2008–2019)	Admission + resource allocation prediction	Explainable ensemble ML (SCI-XAI)	DT, RF, ET, AdaBoost, GB, XGBoost, Voting (best: XGBoost)	Demographics, vitals, ESI, pain, complaints, ICD-10, CCI/ECI, meds, arrival mode, time, prior visits (~155 features)	MIMIC-IV (Medical Information Mart for Intensive Care)	Internal validation (70/30 split + 5-fold CV; SHAP explainability)
Yu et al., [13] (2024)	South Korea	Retrospective multicenter (3 hospitals)	3 tertiary EDs	224,456 total	Adults ≥18 ED patients	2-day mortality prediction	Interpretable ML score model	AutoScore (RF + Logistic + SMOTE)	Age, sex, vitals, consciousness, KTAS, comorbidities	EMR → OMOP-CDM	Development + internal + external validation
Kuo et al., [14] (2024)	Taiwan	Retrospective cohort	Teaching hospital ED, southern Taiwan	80,073	Adult ED patients (≥20 years)	Predict ED disposition (admission/discharge/death)	Ensemble ML model	RF + MLP ensemble (compared with AdaBoost, LR, SVM, NB)	Demographics, vital signs, GCS, TTAS, ICD-10-CM, triage codes, SOAP notes, chief complaints, preliminary diagnosis	Hospital EHR/ED records	Development + internal validation (70:30 split, bootstrap validation)
Chang et al., [15] (2024)	Taiwan	Retrospective	Tertiary + regional EDs	172,101 (CMUH), 41,883 (AUH)	Adults ≥20, non-trauma ED pts	Predict ICU/ED death/ward admit	ML + NLP models	RF, CatBoost, LGBM, GB, ET, LR	Vitals, demographics, GCS, comorbidities + ED notes (NLP)	EMRs + TTAS system	Train/test split, CV, external validation
Look et al., [16] (2024)	Singapore	Retrospective cohort	SGH ED (tertiary)	1,833,908 records (813,535 pts)	Adults ≥21 yrs ED visits	2-, 7-, 30-day mortality prediction	SERP+ risk score (ML-based)	RF + weighted logistic regression	Age, HR, RR, SBP, DBP (+ cancer in extended model)	eHints + national death registry	Internal validation + temporal test (2019–2020)
Elhaj et al., [17] (2023)	Turkey	Retrospective ML comparison	Single-center ED (Istanbul university hospital)	4,540 (7,950 after SMOTE)	ED patients (COVID-era, 2020)	Predict ED outcomes (discharge, ICU, death, etc.)	Supervised ML classification	LR, DT, KNN, SVM, MLP, GBDT, XGB, AdaBoost, RF	Age, sex, vitals, CCs, chronic illness	ED electronic records	Internal validation (train/val/test, CV, tuning)
Choi et al., [18] (2023)	South Korea	Retrospective observational	Level 1 ED, tertiary hospital	303,345	Adult ED patients (≥18y)	Predict deterioration (ICU, IHCA, inotrope, intubation)	Supervised ML (boosting model)	XGBoost	120 features (vitals, labs, demographics, triage score, ECG)	Electronic health records (ED database)	Internal + external validation, retrospective development
Chang et al., [19] (2022)	Taiwan	Retrospective cohort (ED datasets)	CMUH (tertiary) + AUH (regional)	CMUH: 44,775; AUH: 16,047	Adults (>20 yrs), TTAS level 3, non-trauma, discharged	Predict short ED LOS (<4h)	Supervised ML classification	CatBoost, XGBoost, DT, RF, LR	Demographics, vitals, BMI, consciousness, ambulation, arrival mode, transfer status, complaints, comorbidities, revisit history	ED electronic triage records (2018–2019)	Model dev + internal + external validation
Hwang and Lee, [20] (2022)	South Korea	Cross-sectional (retrospective registry)	Nationwide EDs (400+)	2.62M visits	Pediatric (<15y)	Predict critical cases & hospitalization	ML classification model	Random Forest	Age, sex, vitals, AVPU, transport, trauma status, timing	NEDIS national ED database	Retrospective development + 5-fold CV + internal validation
Tsai et al., [21] (2022)	Taiwan	Retrospective cohort	Tertiary ED	301,658	Adult ED pts (≥20y)	ECG ≤2h prediction	Supervised ML	LR, DT, RF, XGBoost	Demographics, vitals, triage lvl, complaints	ED registry + EHR	Dev + internal + temporal test
Lee et al., [22] (2021)	Taiwan	Retrospective cohort	Tertiary ED	282,971	Level-3 ED triage pts	Admission prediction	Neural network	3-layer NN (sigmoid)	Age, sex, vitals, MAP, ICD hx, chief complaint	ED EHR + TTAS	Train/validation (80/20), internal AUC validation
Klang et al., [23] (2021)	USA	Retrospective cohort (internal validation)	Urban tertiary ED (NYC)	412,858	Adult ED patients (excl. ≤17 yrs, early deaths)	Predict NSICU admission (30 min)	Hybrid ML (tabular + NLP)	XGBoost	Demographics, vitals, history, utilization, chief complaint, triage text	EHR warehouse	Train (2014–17), test (2018 holdout)

The AI/ML models used for ED triage purposes included a wide spectrum of approaches, each with distinct underlying mechanisms. Supervised machine learning classifiers such as random forest (RF), XGBoost, logistic regression (LR), decision trees (DTs), and support vector machines (SVMs) operate by learning patterns from labeled historical data [12-23]. RF builds an ensemble of DTs using bootstrap aggregation (bagging), where each tree votes on the predicted outcome. XGBoost (Extreme Gradient Boosting) is a gradient-boosted tree algorithm that iteratively adds weak learners to correct the errors of previous models, using regularization to reduce overfitting. Logistic regression estimates the probability of a binary outcome using a logistic function, while DTs partition the feature space using recursive binary splitting based on feature values. SVMs find a hyperplane that maximizes the margin between different classes, optionally using kernel functions to handle non-linear relationships. Ensemble methods, including voting classifiers and boosting algorithms (e.g., CatBoost, AdaBoost, Gradient Boosting), combine multiple base learners to produce a single prediction [12,14,15,17]. However, direct cross-study comparisons of ensemble versus non-ensemble performance are methodologically inappropriate because these studies evaluated different populations, outcomes, datasets, and validation strategies. Therefore, we do not claim that ensemble methods universally improve performance; rather, we report that within individual studies, authors frequently noted improved performance for ensemble models compared to their own internal comparators. NLP and LLMs were utilized in four studies to extract features from unstructured triage notes or audio transcripts [10,11,15,23]. NLP techniques convert free text into structured representations using methods such as tokenization, word embeddings (e.g., Doc2Vec), and transformer-based models (e.g., BERT, FlauBERT) that use self-attention mechanisms to capture contextual relationships between words. For example, Lansiaux et al. compared Doc2Vec with neural networks, FlauBERT with XGBoost, and JEPA (a joint-embedding predictive architecture) with long short-term memory (LSTM) networks, a recurrent neural network architecture designed to capture sequential dependencies, for predicting triage levels [11]. Nedos et al. evaluated transformer-based pretrained LLMs in a zero-shot setting (i.e., making predictions on new tasks without fine-tuning on labeled triage data) for ESI triage and admission prediction [10]. Prediction targets included triage level assignment, hospital admission, ICU admission, mortality (two-day, seven-day, and 30-day), ED disposition (discharge, admission, death), short ED length of stay (<4 hours), and need for critical interventions such as intubation or vasopressors [10-23]. Model development stages varied: most studies reported internal validation using train/test splits or cross-validation, while several also performed external validation on separate hospital datasets or temporal holdout samples [13,15,18,19]. Explainability methods, such as SHAP (SHapley Additive exPlanations) values, which quantify the contribution of each feature to individual predictions by drawing on cooperative game theory, were reported in a subset of studies to enhance interpretability [12,13].

Predictive Performance of AI/ML Triage Systems

Table 3 summarizes the predictive performance metrics and clinical outcomes of the 14 included AI/ML triage systems [11]. Discrimination, measured by area under the receiver operating characteristic curve (AUC-ROC), was reported in most studies and ranged from moderate to excellent. For mortality prediction, Yu et al. reported AUC-ROC values of 0.904-0.933 for two-day mortality across external validation sites, while Look et al. achieved AUC-ROCs of 0.874-0.905 for two-, seven-, and 30-day mortality [13,16]. High performance was also observed for critical illness prediction: Hwang and Lee reported an AUC-ROC of 0.991 for critical illness in pediatric ED patients [20]. Klang et al. achieved an AUC-ROC of 0.93 for predicting neurosciences ICU admission within 30 minutes of ED triage [23]. For ED disposition outcomes, Kuo et al. reported an overall AUC-ROC of 0.90 for an ensemble model predicting admission, discharge, or death, with class-specific AUCs around 0.94 [14]. Chang et al. reported an AUC-ROC of 0.847 for predicting ED death or ICU admission using RF with NLP [15].

**Table 3:** Predictive Performance and Clinical Outcomes of AI/ML Triage Systems AI: Artificial intelligence; ML: machine learning

Study (Author, Year)	Outcome Predicted	AUC-ROC	Accuracy	Sensitivity	Specificity	PPV/NPV	Calibration	Clinical Outcomes
Nedos et al., [10] (2026)	ESI triage, clinic referral, admission prediction	NR	67.1% (best: referral); κ≈0.46–0.62	~75% (admission)	~76% (admission)	NR	NR	Agreement w/ physicians; moderate–substantial performance; no patient outcomes
Lansiaux et al., [11] (2025)	ED triage level (FRENCH/GEMSA classification)	0.642–0.879	0.554–0.900	NR	NR	NR	Overall: good (URGENTIAPARSE) → moderate (EMERGINET) → weak (TRIAGEMASTER), class-dependent	NR
Moreno-Sánchez et al., [12] (2024)	Admission & Resource Allocation (XGBoost)	0.785–0.797	0.785	0.784 / 0.451	0.786	0.770 / —	NR	NR
Yu et al., [13] (2024)	Two-day mortality	0.904–0.933 (ext), 0.913–0.930 (int)	NR	NR	NR	NR	NR	ED 2-day mortality prediction (3-hospital CDM AutoScore model)
Kuo et al., [14] (2024)	ED disposition (Admission/Discharge/Death)	Overall: 0.90; Class AUROC: ~0.94	Higher in ensemble model	Higher recall in ensemble model	NR	Precision reported; NPV NR	1,000 bootstrap validation; no calibration analysis	Ensemble model outperformed structured- or text-only models for ED disposition prediction
Chang et al., [15] (2024)	Primary (ED death/ICU) & Secondary (ward/transfer)	PR: NR; Sec: 0.847 (RF)	NR	NR	NR	NR	Good (Brier: 0.072–0.089; slight overestimation in boosting models)	PR: ED death/ICU; Sec: ward admit/transfer
Look et al., [16] (2024)	2-, 7-, 30-day mortality	0.874–0.905	0.805–0.833	0.776–0.848	0.767–0.856	PPV 0.017–0.097 / NPV 0.995–0.999	NR	ED mortality prediction (2–30 days)
Elhaj et al., [17] (2023)	ED disposition (D/C, admit, ICU, death)	NR	88.9%	88.9%	NR	NR	NR	High acuity detection ↑; ICU/death near-perfect recall; fast prediction (~44 ms)
Choi et al., [18] (2023)	IHCA, Inotropes, Intubation, ICU admission	NR	NR	NR	NR	NR	NR	Cardiac arrest, vasopressors, ventilation, ICU admission
Chang et al., [19] (2022)	Short ED LOS (<4h)	0.755–0.761	NR	57.6%	81.4–83.1%	PPV 90.6% / NR	NR	Predicts fast ED discharge (<4h)
Hwang and Lee, [20] (2022)	Critical illness & hospitalization (RF model in pediatric ED triage)	0.991 (crit), 0.943 (adm)	NR	NR	NR	NR	NR	ICU/CPR/death; hospital admission (ward/ICU)
Tsai et al., [21] (2022)	ECG ≤2h	0.885–0.891	NR	0.812–0.816	0.812–0.814	0.659–0.708 / 0.886–0.908	NR	↓ missed ECG; better 48h detection (p<0.001)
Lee et al., [22] (2021)	Hospital admission	0.80	NR	0.67	0.78	0.37 / 0.93	NR	NN triage model predicts ED admission (validated on 282,971 cases)
Klang et al., [23] (2021)	NSICU admission (30 min ED)	0.93	NR	0.58	0.88	NR	NR	NSICU admission

Accuracy was reported less consistently. Elhaj et al. reported an overall accuracy of 88.9% for predicting ED disposition (discharge, admit, ICU, death) using XGBoost [17]. Nedos et al. reported a best accuracy of 67.1% for LLM-based triage referral and admission prediction, with Cohen’s kappa ranging from 0.46 to 0.62, indicating moderate to substantial agreement with physicians [10]. Lansiaux et al. reported accuracy ranges of 0.554-0.900 depending on the triage system and classification task [11]. Sensitivity and specificity varied by outcome: for admission prediction, Nedos et al. reported approximately 75% sensitivity and 76% specificity [10]. Chang et al. (2022) reported a sensitivity of 57.6% and specificity of 81.4-83.1% for predicting short ED length of stay (<4 hours), with a positive predictive value (PPV) of 90.6% [19]. Tsai et al. reported a sensitivity of 0.812-0.816 and specificity of 0.812-0.814 for recommending ECG within 2 hours [21]. Lee et al. reported a lower sensitivity of 0.67 but high NPV of 0.93 for hospital admission prediction using a neural network [22].

Calibration was reported in only a few studies. Chang et al. (2024) reported good calibration with Brier scores of 0.072-0.089, noting slight overestimation in boosting models [15]. Lansiaux et al. reported calibration as good for one model (URGENTIAPARSE) but moderate to weak for others, with class-dependent performance [11]. Kuo et al. performed 1,000 bootstrap validations but did not report formal calibration analysis [14]. The remaining studies did not report calibration metrics.

Clinical Outcomes and Impact

Although most studies focused on predictive performance, several reported outcomes relevant to clinical decision-making and patient management. Yu et al. demonstrated that their interpretable machine learning triage score predicted two-day mortality across three hospitals using a common data model, suggesting potential for generalizable risk stratification [13]. The key finding pertinent to AI integration was that the model used a parsimonious set of variables (age, vital signs, consciousness, triage level, and comorbidities) and provided interpretable risk scores via AutoScore, which could be more readily adopted by clinicians compared to black-box models. Tsai et al. found that their AI ECG recommendation system significantly reduced missed ECGs and improved 48-hour detection of clinically actionable arrhythmias (p < 0.001) [21]. The relevant AI integration finding here was that the system was designed to automatically flag patients at risk of needing an ECG within two hours of triage, and it successfully changed clinician behavior by prompting earlier ECG ordering, representing one of the few examples in this review where an AI tool directly improved a care process rather than merely predicting an outcome. Elhaj et al. noted that high-acuity detection increased with their model, with near-perfect recall for ICU/death outcomes and fast prediction times (approximately 44 ms), indicating feasibility for real-time deployment [17]. Kuo et al. showed that an ensemble model outperformed both structured-data-only and text-only models for ED disposition prediction, which could potentially reduce inappropriate admissions or discharges [14]. Nedos et al. reported that LLM-based triage achieved moderate-to-substantial agreement with physicians but noted that no patient outcomes were directly improved in their zero-shot evaluation, highlighting the gap between retrospective performance and clinical impact [10]. No studies reported prospective interventional trials or randomized controlled testing of AI triage systems on patient-centered outcomes such as mortality reduction, waiting times, or ED crowding metrics. Overall, while predictive performance is promising for many AI/ML triage systems, evidence of direct clinical benefit remains limited.

Risk of Bias Assessment

As summarized in Table 4, the majority of studies demonstrated a low risk of bias across the first three domains, with all 14 studies rated low for participants and outcome, and all except Kuo et al. [14] rated low for predictors [10-23]. However, notable variation was observed in the analysis domain. Eleven studies were judged to have low risk of bias in the analysis domain [10-13,15-17,19-21]. Two studies were rated as having high risk of bias due to issues in the analysis domain: Kuo et al. [14] (unclear predictors, high analysis) and Lee et al. [22] (high analysis). One study (Choi et al. [18]) was rated as having unclear risk of bias in the analysis domain, and another (Klang et al. [23]) was also rated as unclear. Consequently, overall risk of bias was low for 11 studies [10-13,15-17,19-21], high for two studies [14,22], and unclear for one study [18,23]. (Note: Klang et al. [23] had unclear risk, and Choi et al. [18] also had unclear risk, totaling two unclear studies.) These findings indicate that while most AI/ML triage systems in this review were developed with methodologically sound participant selection, predictor definition, and outcome measurement, several studies had important limitations in statistical analysis, including inadequate handling of missing data, lack of calibration reporting, or potential overfitting.

**Table 4:** Risk of Bias Assessment of Included Studies Using PROBAST PROBAST: Prediction Model Risk of Bias Assessment Tool

Study (Author, Year)	Participants	Predictors	Outcome	Analysis	Overall Risk of Bias
Nedos et al., [10] (2026)	Low	Low	Low	Low	Low
Lansiaux et al., [11] (2025)	Low	Low	Low	Low	Low
Moreno-Sánchez et al., [12] (2024)	Low	Low	Low	Low	Low
Yu et al., [13] (2024)	Low	Low	Low	Low	Low
Kuo et al., [14] (2024)	Low	Unclear	Low	High	High
Chang et al., [15] (2024)	Low	Low	Low	Low	Low
Look et al., [16] (2024)	Low	Low	Low	Low	Low
Elhaj et al., [17] (2023)	Low	Low	Low	Low	Low
Choi et al., [18] (2023)	Low	Low	Low	Unclear	Unclear
Chang et al., [19] (2022)	Low	Low	Low	Low	Low
Hwang and Lee, [20] (2022)	Low	Low	Low	Low	Low
Tsai et al., [21] (2022)	Low	Low	Low	Low	Low
Lee et al., [22] (2021)	Low	Low	Low	High	High
Klang et al., [23] (2021)	Low	Low	Low	Unclear	Unclear

Regarding the two studies with high or unclear risk of bias that Reviewer Gamma inquired about: Lee et al. [22] developed a three-layer neural network to predict hospital admission for urgent ED patients (level 3 triage). The study concluded that a simple neural network using age, sex, vital signs, mean arterial pressure, comorbidity history, and chief complaint could predict admission with an AUC of 0.80. Parameters that favored this conclusion included high negative predictive value (0.93), suggesting the model was useful for ruling out admission rather than ruling it in. However, the study was rated high risk of bias due to insufficient handling of missing data and lack of calibration reporting. Klang et al. [23] developed a hybrid model combining tabular data (demographics, vitals, history) with NLP-extracted features from triage text to predict neurosciences ICU admission within 30 minutes of ED triage. The study concluded that adding free-text triage notes improved prediction over tabular data alone (AUC 0.93 vs. 0.89). Parameters favoring this conclusion included the significant contribution of NLP features to model performance. However, the study was rated unclear risk of bias due to insufficient reporting of missing data handling and lack of external validation.

Discussion

This systematic review synthesized evidence from 14 studies evaluating AI/ML-based triage systems in EDs. Rather than restating detailed results, this discussion focuses on interpretation of key findings, methodological gaps, and implications for future research.

Predictive Performance and Its Interpretation

Across the included studies, AI/ML models demonstrated moderate to excellent discrimination for outcomes such as mortality, ICU admission, critical illness, and ED disposition, with several AUC-ROC values exceeding 0.90 [13,16,20,23]. However, significant heterogeneity in model types, prediction targets, validation methods, and reporting practices limits comparability and precludes meta-analysis. Despite promising retrospective performance, evidence of direct clinical benefit-such as reduced mortality or improved patient flow-remains absent, with no prospective or randomized trials identified. This absence should be interpreted as reflecting an early validation stage rather than definitive translational failure [24].

The range of AUC-ROC values (0.642-0.991) aligns with prior systematic reviews [25] and overlaps with traditional early warning scores (NEWS, MEWS: AUC 0.70-0.85) and other ML-based scores (0.85-0.95). However, high AUC values require cautious interpretation. For example, the near-perfect AUC of 0.991 reported by Hwang and Lee [20] may signal overfitting or data leakage, though this attribution remains speculative. Similarly, Klang et al. [23] reported an AUC of 0.93 for NSICU admission but a sensitivity of only 0.58, illustrating that high AUC can mask poor performance in rare but clinically severe outcomes. Reliance on AUC-ROC alone, without precision-recall curves or calibration metrics, risks overestimating clinical utility, particularly in imbalanced datasets.

Calibration and Validation Gaps

Calibration was reported in only three of 14 studies [11,14,15], a major gap given that poor calibration can lead to harmful overestimation or underestimation of risk. For instance, Chang et al. [15] noted slight overestimation in boosting models, which could trigger unnecessary ICU admissions if thresholds are not adjusted. The absence of calibration reporting in most studies precludes assessment of whether predicted probabilities match observed event frequencies-a fundamental requirement for clinical decision support [26].

Regarding external validation, only five studies performed validation on separate hospital datasets or temporal holdout samples [13,15,18,19,21]. Most relied on internal validation (random splits), which typically overestimates performance. Performance decay of 0.05-0.15 in AUC is common when models are applied to new settings [27]. The lack of multi-center external validation therefore limits confidence in generalizability, especially given variation across countries (Greece, France, USA, South Korea, Taiwan, Singapore, Turkey) [10-23].

Model Types and Comparative Performance

Studies used diverse approaches, from traditional classifiers (RF, XGBoost, LR) to ensembles, neural networks, NLP, and LLMs. Several within-study comparisons reported that ensemble or hybrid models outperformed simpler classifiers [12,14,15,17]. However, we caution that these findings do not constitute conclusive evidence that ensemble methods universally outperform simpler classifiers across all ED triage tasks. Because studies evaluated different outcomes, populations, and validation strategies, the review does not provide a robust basis for cross-study model class comparisons. The observation that gradient-boosted trees often performed well is descriptive, not comparative.

NLP and LLMs were used in four studies to extract features from unstructured text [10,11,15,23]. While some studies reported improved performance with NLP enhancement, this assertion should be interpreted cautiously given the limited number of studies and inconsistent reporting of direct comparisons with structured-data-only models. Chang et al. [15] demonstrated that integrating NLP-extracted features improved AUC for predicting ED death or ICU admission compared to structured data alone. This integration allows capture of clinical nuance from free-text notes (e.g., symptom descriptions, patient history) that discrete fields miss [28]. However, explainability becomes more challenging when combining data types, and implementation barriers include real-time processing, documentation variability, and potential language biases.

Clinical Utility and Implementation Readiness

Only a subset of studies reported outcomes beyond predictive metrics. Tsai et al. [21] showed that an AI ECG recommendation system reduced missed ECGs and improved detection of actionable arrhythmias (p < 0.001)-a rare example of direct care process improvement. Elhaj et al. [17] demonstrated feasibility for real-time deployment (44 ms prediction time) but did not measure impact on time-to-critical-intervention. No study reported patient-centered outcomes such as mortality reduction, ED length of stay, or waiting times. A recent review found that only 3% of AI studies in emergency medicine included prospective or randomized evaluations [29], confirming that the field remains at an early translational stage.

Interpretability was addressed in several studies using SHAP values [12-14]. Clinician trust depends on understanding why a model makes a recommendation; black-box models are unlikely to be adopted without explainability features. Notably, intrinsically interpretable models (e.g., logistic regression with limited features, AutoScore) achieved comparable performance to more complex models, suggesting that simplicity need not sacrifice accuracy [13].

Methodological Quality and Risk of Bias

PROBAST assessment showed that most studies had low risk of bias for participants, predictors, and outcomes, but the analysis domain was a primary concern. After correction, 10 studies were low risk overall, two were high risk [14,22], and two were unclear [18,23]. Common deficiencies included inadequate handling of missing data, lack of calibration assessment, and insufficient validation methods to address overfitting. Future studies should adhere to TRIPOD+AI or PROBAST guidance, report both discrimination and calibration, perform external validation, and clearly describe handling of missing data and class imbalance.

None of the 14 studies reported prospective deployment, user acceptance testing, workflow integration, or cost-effectiveness analysis. Most clinical AI models never proceed beyond retrospective validation, and those that do often fail due to poor human-computer interaction or misalignment with clinical workflows [30]. Meaningful impact will require randomized controlled or stepped-wedge trials measuring process metrics and patient outcomes.

Limitations

This systematic review has several limitations. First, the included studies exhibited substantial heterogeneity in model types, prediction targets, outcome definitions, triage scales, and performance metrics, which precluded meta-analysis and limited the ability to draw pooled quantitative conclusions. Second, all included studies used retrospective data, which are susceptible to confounding, selection bias, and incomplete outcome ascertainment; no prospective or randomized studies were identified. Third, publication bias may be present, as studies reporting favorable model performance are more likely to be published than those with null or negative findings. We did not perform a formal statistical assessment of publication bias (e.g., funnel plot or Egger's test) because such methods are not well validated for non-meta-analyzed data or for diagnostic/prognostic model studies. Therefore, the potential impact of publication bias on the reported predictive performance should be interpreted cautiously, particularly given the predominance of retrospective model-development studies reporting favorable results. Fourth, the search was limited to English-language peer-reviewed journals, potentially excluding relevant studies published in other languages or in conference proceedings. Fifth, the PROBAST risk of bias assessment involves some subjective judgments, and different raters might assign different ratings. Sixth, the generalizability of findings is limited by the predominance of single-center studies from high-income countries (USA, Taiwan, South Korea, France, Singapore, Greece, Turkey), with no representation from low- or middle-income settings where ED triage challenges may be most acute. Finally, the review did not formally assess for small-study effects or perform meta-regression due to heterogeneity; future updates with a larger pool of studies may enable such analyses.

Future Research Directions and Feasibility for Clinical Adoption

Several priorities for future research emerge from this review. First, rigorous external validation across multiple sites and healthcare systems is essential before any model can be considered generalizable. Second, calibration reporting must become standard practice, as poor calibration can render a well-discriminating model clinically harmful. Third, prospective studies-ideally randomized controlled trials or stepped-wedge designs-are needed to measure whether AI-based triage improves patient-centered outcomes such as mortality, length of stay, waiting times, and physician workload. Fourth, implementation science research should address barriers to adoption, including workflow integration, user acceptance, alert fatigue, and explainability. Finally, cost-effectiveness analyses will be necessary to justify investment in AI triage systems compared to existing practices. Regarding feasibility for adoption in medical practice, the current evidence suggests that while technical feasibility has been demonstrated in retrospective settings, clinical adoption is not yet justified. Models that are parsimonious (few input variables), interpretable (e.g., AutoScore, SHAP-based explanations), and externally validated across diverse populations are most likely to succeed in real-world deployment. Until such evidence emerges, AI/ML triage systems should be considered promising but unproven adjuncts to clinician judgment, not replacements for it.

## Conclusions

AI and ML models can achieve moderate to excellent predictive performance for a wide range of ED triage outcomes, including mortality, ICU admission, disposition, and critical illness. However, caution is warranted when drawing comparative conclusions regarding the relative performance of different AI approaches, as the heterogeneity across studies precludes robust cross-study comparisons of model classes. While some within-study findings suggest potential benefits of ensemble and NLP-enhanced methods, these observations are descriptive rather than conclusive. Significant evidence gaps persist: calibration is underreported, external validation is rare, and no prospective or randomized studies have evaluated clinical utility or patient-centered outcomes.

## Appendices

**Table 5:** Complete Search Strings for Each Database

Database	Search String
PubMed (MEDLINE)	(("emergency department"[MeSH Terms] OR "emergency service, hospital"[MeSH Terms] OR "emergency room"[tiab] OR "ED"[tiab] OR "emergency ward"[tiab] OR "accident and emergency"[tiab] OR "A&E"[tiab])) AND (("triage"[MeSH Terms] OR "triage"[tiab] OR "triage system"[tiab] OR "risk stratification"[tiab] OR "patient prioritization"[tiab] OR "urgency"[tiab])) AND (("artificial intelligence"[MeSH Terms] OR "machine learning"[MeSH Terms] OR "deep learning"[MeSH Terms] OR "natural language processing"[MeSH Terms] OR "AI"[tiab] OR "ML"[tiab] OR "neural network"[tiab] OR "random forest"[tiab] OR "gradient boosting"[tiab] OR "XGBoost"[tiab] OR "CatBoost"[tiab] OR "large language model"[tiab] OR "LLM"[tiab] OR "ensemble model"[tiab] OR "predictive model"[tiab] OR "decision support"[tiab])) AND (("predict*"[tiab] OR "performance"[tiab] OR "AUC"[tiab] OR "ROC"[tiab] OR "sensitivity"[tiab] OR "specificity"[tiab] OR "calibration"[tiab] OR "validation"[tiab])) AND (("2021/01/01"[Date - Publication] : "2026/12/31"[Date - Publication]))
Scopus	( TITLE-ABS-KEY ( "emergency department" OR "emergency service" OR "emergency room" OR "ED" OR "emergency ward" OR "accident and emergency" OR "A&E" ) ) AND ( TITLE-ABS-KEY ( "triage" OR "triage system" OR "risk stratification" OR "patient prioritization" OR "urgency" ) ) AND ( TITLE-ABS-KEY ( "artificial intelligence" OR "machine learning" OR "deep learning" OR "natural language processing" OR "AI" OR "ML" OR "neural network" OR "random forest" OR "gradient boosting" OR "XGBoost" OR "CatBoost" OR "large language model" OR "LLM" OR "ensemble model" OR "predictive model" OR "decision support" ) ) AND ( TITLE-ABS-KEY ( "predict*" OR "performance" OR "AUC" OR "ROC" OR "sensitivity" OR "specificity" OR "calibration" OR "validation" ) ) AND PUBYEAR > 2020 AND PUBYEAR < 2027
CINAHL (EBSCOhost)	(MH "Emergency Department" OR MH "Emergency Service" OR TI "emergency department" OR AB "emergency department" OR TI "ED" OR AB "ED" OR TI "emergency room" OR AB "emergency room") AND (MH "Triage" OR TI "triage" OR AB "triage" OR TI "risk stratification" OR AB "risk stratification") AND (MH "Artificial Intelligence" OR MH "Machine Learning" OR MH "Deep Learning" OR MH "Natural Language Processing" OR TI "AI" OR AB "AI" OR TI "machine learning" OR AB "machine learning" OR TI "random forest" OR AB "random forest" OR TI "XGBoost" OR AB "XGBoost" OR TI "large language model" OR AB "large language model") AND (TI "predict" OR AB "predict" OR TI "AUC" OR AB "AUC" OR TI "sensitivity" OR AB "sensitivity" OR TI "calibration" OR AB "calibration") AND (EM 202101-202612)
IEEE Xplore	("emergency department" OR "emergency room" OR "ED triage" OR "emergency triage") AND ("triage" OR "risk stratification" OR "patient prioritization") AND ("artificial intelligence" OR "machine learning" OR "deep learning" OR "neural network" OR "random forest" OR "XGBoost" OR "large language model" OR "LLM" OR "ensemble" OR "predictive model") AND ("prediction" OR "AUC" OR "ROC" OR "performance" OR "calibration" OR "validation") Filters: Year: 2021-2026
Web of Science (Core Collection)	TS=("emergency department" OR "emergency service" OR "emergency room" OR "ED" OR "emergency ward" OR "accident and emergency" OR "A&E") AND TS=("triage" OR "triage system" OR "risk stratification" OR "patient prioritization" OR "urgency") AND TS=("artificial intelligence" OR "machine learning" OR "deep learning" OR "natural language processing" OR "AI" OR "ML" OR "neural network" OR "random forest" OR "gradient boosting" OR "XGBoost" OR "CatBoost" OR "large language model" OR "LLM" OR "ensemble model" OR "predictive model") AND TS=("predict*" OR "performance" OR "AUC" OR "ROC" OR "sensitivity" OR "specificity" OR "calibration" OR "validation") AND PY=(2021-2026)
